# Evidence for Structural and Functional Alterations of Frontal-Executive and Corticolimbic Circuits in Late-Life Depression and Relationship to Mild Cognitive Impairment and Dementia: A Systematic Review

**DOI:** 10.3389/fnins.2020.00253

**Published:** 2020-04-17

**Authors:** Neda Rashidi-Ranjbar, Dayton Miranda, Meryl A. Butters, Benoit H. Mulsant, Aristotle N. Voineskos

**Affiliations:** ^1^Institute of Medical Science, University of Toronto, Toronto, ON, Canada; ^2^Campbell Family Mental Health Institute, Centre for Addiction and Mental Health, Toronto, ON, Canada; ^3^Department of Psychiatry, University of Pittsburgh School of Medicine, Pittsburgh, PA, United States; ^4^Department of Psychiatry, University of Toronto, Toronto, ON, Canada

**Keywords:** late-life depression, mild cognitive impairment, Alzheimer's disease, frontal-executive, corticolimbic, diffusion-tensor imaging, resting-state functional MRI, brain network

## Abstract

Depression is a risk factor for developing Alzheimer's disease and Related Dementia (ADRD). We conducted a systematic review between 2008 and October 2018, to evaluate the evidence for a conceptual mechanistic model linking depression and ADRD, focusing on frontal-executive and corticolimbic circuits. We focused on two neuroimaging modalities: diffusion-weighted imaging measuring white matter tract disruptions and resting-state functional MRI measuring alterations in network dynamics in late-life depression (LLD), mild cognitive impairment (MCI), and LLD+MCI vs. healthy control (HC) individuals. Our data synthesis revealed that in some but not all studies, impairment of both frontal-executive and corticolimbic circuits, as well as impairment of global brain topology was present in LLD, MCI, and LLD+MCI vs. HC groups. Further, posterior midline regions (posterior cingulate cortex and precuneus) appeared to have the most structural and functional alterations in all patient groups. Future cohort and longitudinal studies are required to address the heterogeneity of findings, and to clarify which subgroups of people with LLD are at highest risk for developing MCI and ADRD.

## Introduction

### Alzheimer's Disease and Related Dementias

The number of individuals with Alzheimer's disease and related dementia (ADRD) is expected to rise to 82 million in 2030 and 152 million in 2050 (WHO Fact Sheet, 2019). One in every 2–3 people over the age of 85 will develop ADRD (Hebert et al., 2013). In the 2011 guideline, “Alzheimer's dementia” is referred to what was “Alzheimer's disease” under the 1984 guidelines. Alzheimer's dementia is referring to the dementia stage of the Alzheimer's disease continuum that starts with initial brain changes leading to cognitive and physical symptoms over years (Association Alzheimer's, 2018). Delaying disease onset or progression by even 1 year can lead to a significant reduction of the global burden of disease (Brookmeyer et al., 2007). Absent a breakthrough drug, the “lowest hanging fruit” for delay or prevention of ADRD consists of several modifiable risk factors. These include depression, diet, type 2 diabetes mellitus, midlife hypertension, current smoking, cognitive inactivity, physical inactivity, and traumatic brain injury (Daviglus et al., 2010; Baumgart et al., 2015; Xu et al., 2015; Clare et al., 2017).

### Depression and ADRD: The Epidemiological Connection

Cohort studies and meta-analyses have shown that late life depression (LLD) increases the risk for dementia in general, and AD in particular (Jorm, 2001; Ownby et al., 2006; Diniz et al., 2013). More specifically, depression is a risk factor for the progression from normal cognition to MCI, and from MCI to dementia (Diniz et al., 2013; Brailean et al., 2017).

Depression is currently the leading cause of disability and disease worldwide (WHO Fact Sheet, 2019). With the increased rates of depression and the aging of the world's population (Compton et al., 2006) the incidence of depression in late life will rise (Chapman and Perry, 2008; Zivin et al., 2013). Older adults with depression develop mild cognitive impairment (MCI)—an intermediate stage between normal cognition and AD with a ratio of 6–15% per year (Farias et al., 2009; Petersen et al., 2009). More strikingly, one-third to one-half of older individuals with depression have a concomitant diagnosis of MCI, far higher than the general population (Butters et al., 2004; Bhalla et al., 2006; Barnes and Yaffe, 2011). However, the relative risk among studies varies. Discrepancies may be due to study population differences or methodologic differences between the various studies (Byers and Yaffe, 2011; Köhler et al., 2011). Alternatively, whether different age of onset of depression [i.e., early-onset depression (EOD)] with an age of onset below 60, as opposed to late-onset depression (LOD; Brodaty et al., 2001; Hashem et al., 2017) would influence the progression to AD remains to be elucidated (Edwards et al., 2019). For instance, a large-scale retrospective cohort study reported a 20 and 70% adjusted hazard of dementia in midlife and late-life depressive symptoms respectively (Barnes et al., 2012). Furthermore, the risk may vary due to depression severity, or its successful treatment (Almeida et al., 2017).

### Cognitive Impairment in LLD, MCI, and AD Is Shared Across Memory and Executive Function Domains

Cognitive impairment, specifically in executive function and information processing speed corresponding to the frontal-executive circuit, and in episodic memory corresponding to the corticolimbic circuit, are common findings among LLD, MCI, and AD (Butters et al., 2008; Koenig et al., 2015; Liao et al., 2017). Episodic memory, executive function, and processing speed, are the main impaired cognitive domains in individuals with aMCI (Alexopoulos et al., 2012; Bai et al., 2012), LLD+MCI, and AD (Geerlings et al., 2008; Li H. J. et al., 2015). This impairment usually occurs to a greater extent in individuals with aMCI, LLD+MCI, and AD, relative to individuals with LLD (Bai et al., 2012; Wang L. et al., 2012; Li et al., 2014; Shimoda et al., 2015). In studies using a comprehensive neuropsychological battery, cognitive impairment in LLD, compared to HC, was observed especially in episodic memory, executive function, and processing speed (Bai et al., 2012; Koenig et al., 2015; Chen et al., 2016; Liao et al., 2017; Li W. et al., 2017). However, other cognitive findings in LLD are variable. For instance, a longitudinal study has reported poor cognitive performance in individuals with depression at baseline, compared to a HC group, and greater decline in episodic memory, attention-working memory, and executive function after both 3 and 12 months. Non-remitters had greater decline in executive function after 12 months (Riddle et al., 2017). Furthermore, LLD compared to HC groups have shown to have impairment in executive function, but no difference in verbal and visuospatial memory (Dybedal et al., 2013), or impairment in attention, and memory functions, but no difference in total cognitive function, processing speed, and executive function (Yue et al., 2015). Other studies have found no differences in the cognitive profile of LLD and HC groups (Alexopoulos et al., 2012), at baseline or over 1 year follow up (Li X. et al., 2017). The discrepancies may be due to differences in the cognitive tests that were used in these studies. A recent meta-analysis synthesized the evidence on cognitive impairment associated with depression in older adults and found that LLD was significantly associated with global cognitive impairment (John et al., 2018). However, the majority of studies in this meta-analysis had used the Mini-Mental State Examination (MMSE), a cognitive screening test that has ceiling effects in LLD and lacks sensitivity to subtle changes in specific cognitive domains (Tombaugh and McIntyre, 1992; Rajji et al., 2009). Thus, this meta-analysis could not report how LLD influences impairment in different cognitive domains, such as memory, executive function, or information processing speed (John et al., 2018).

### The Potential Mechanisms Linking LLD and ADRD

In 2008, we proposed a conceptual model of the potential etiopathological mechanisms linking late-life depression (LLD) and ADRD (Butters et al., 2008). We hypothesized that three main pathways might explain the neurobiology linking depression with ADRD: a vascular pathway; aligning by and large with executive dysfunction, an inflammation pathway; aligning largely with episodic memory impairment, and an amyloid pathway, serving to reduce time to clinical dementia symptoms. The “vascular hypothesis” suggests that cerebrovascular disease (CVD) may lead to structural damage, particularly to frontal-striatal or frontal-executive circuits (Alexopoulos, 2003; Diniz et al., 2013) that in turn leads to executive dysfunction in both depression and ADRD (Alexopoulos et al., 1997; Krishnan et al., 1997). The “inflammation hypothesis” posits that the higher levels of cortisol (due to hyperactivity of the hypothalamic-pituitary-adrenal axis) (Paul, 2001) and proinflammatory cytokines (Diniz et al., 2010; Hermida et al., 2012) associated with depression lead especially to hippocampal volume loss (i.e., damage to the corticolimbic circuit) and cognitive deficits (e.g., episodic memory) in older adults (Lee et al., 2007; Peavy et al., 2007). Finally, we proposed that increased amyloid production seen in preclinical studies and in pre- and post-mortem human studies of depression (Osorio et al., 2014; Mahgoub and Alexopoulos, 2017) may lead to increases in ADRD neuropathology. While 50% of individuals with LLD present with beta amyloid within the AD range (Butters et al., 2008), recent data do not support a role for amyloid in the depression-dementia link (Diniz et al., 2015; Nascimento et al., 2015; Morin et al., 2019). Each of these processes can independently or collectively contribute to brain injury burden, lower cognitive reserve, and lead to neurodegeneration and cognitive loss. This conceptualized “multiple pathways model” and associated mechanisms (some fixed, some progressive) may lead to heterogeneous cognitive outcomes—i.e., “normal” cognition, MCI, Alzheimer's dementia (AD), vascular Dementia (VaD), or mixed AD with cerebrovascular disease.

### Progress in the Assessment of Brain Circuits and Networks

We can now interrogate brain circuits through the use of newer structural MRI techniques and identify networks with functional MRI. Unlike the original MRI studies of LLD or ADRD, these newer methods can use broadly adopted acquisition sequences, large sample sizes, and longitudinal follow-up. In contrast to the earlier focus on specific brain regions, recent studies interrogating brain circuits or networks can test whether there is overlap in the neural circuits implicated in LLD and in MCI or dementia. Thus, we conducted a systematic review of brain circuits reported to be impaired in LLD to complement and extend the mechanistic model proposed by Butters et al. (2008) more than 10 years ago.

### Objectives and Hypotheses

We conducted a review to evaluate the evidence in overlap in alteration of brain circuits and networks and the associated cognitive impairment between depression and ADRD. We focused on two neuroimaging modalities: diffusion-weighted imaging (DWI) measuring white matter tract disruptions and resting-state functional MRI (rs-fMRI) measuring alterations in network dynamics. Based on the model proposed by Butters et al. (2008) and subsequent studies (Bhalla et al., 2009; Wu et al., 2011; Andreescu et al., 2013; Diniz et al., 2013, 2016; Boccia et al., 2015; Smagula et al., 2015), we hypothesized that evidence would support similar structural and functional disruptions in two circuits: (i) the frontal-executive circuit subserving executive function and the executive control network (ECN) and (ii) the corticolimbic circuit subserving episodic memory and the default mode network (DMN) in LLD and MCI or ADRD. We also explored whether the evidence would suggest similar or different structural or functional changes mechanisms in the two groups of individuals with LLD: those with early-onset depression (EOD; age of onset before 60 recurring in late life and those with late-onset depression (LOD; age of onset after 60) (Brodaty et al., 2001; Hashem et al., 2017).

### Overview of Identifying Structural Brain Circuit Using Diffusion-Weighted Imaging (DWI)

DWI estimates water molecule diffusion and their directionality. The most common DWI measures are fractional anisotropy (FA), a potential marker of axonal structural integrity; and mean diffusivity (MD) a reflection of rotationally invariant variations within the intracellular and extracellular space (Winston, 2012). Pathological damage can be detected by decreased FA and increased MD. FA and MD can be measured locally using a predefined region of interest (ROI) analysis or globally with voxel-wise tract-based spatial statistics (TBSS) (Smith et al., 2006). Tractography is a DWI-based computational reconstruction method for the mapping of discrete fiber tracks, using defined originating regions (“seeds”) and statistical procedures; it can follow the trajectories of white-matter tracts *in vivo* and infer the underlying structural connectome of the human brain (Jbabdi et al., 2015). DWI can provide other measures to indirectly index white matter health or pathology; however, this review focused on the most commonly used FA and MD metrics.

### Overview of Identifying Functional Brain Network Using Resting-State fMRI (rs-fMRI)

Brain activity and network connectivity at rest can be measured using rs-fMRI. While a variety of analytical strategies are available to study resting-state network connectivity, this review focuses on three common approaches: (1) seed-based analysis: a hypothesis-driven approach where an a priori seed region of interest is initially selected and a brain connectivity map is calculated by detecting temporal correlation, i.e., synchronous co-activation, between that seed and all other regions in the brain (Lee M. H. et al., 2013); (2) an independent component analysis (ICA): a non-hypothesis, data-driven and more complex approach that evaluates the whole brain and decomposes it into a set of independent components, each depicted as a functional map (Van Den Heuvel et al., 2009; Wang N. et al., 2012); and (3) regional homogeneity (ReHo): an approach that evaluates the similarity or synchronization between the time series of a given voxel and its nearest neighbors (Zang et al., 2004).

Additionally, some DWI and rs-fMRI studies use more advanced data analysis techniques, including network and graph theory analyses, which quantify whole brain topology and its organization features (Bullmore and Sporns, 2009).

## Methods

### Search Strategy and Data Sources

**Figure 1:**
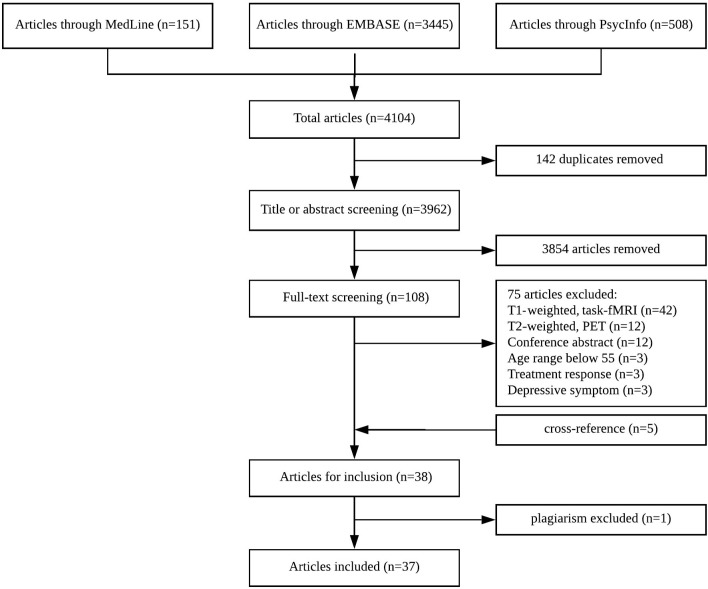
PRISMA flowchart.

A systematic literature search was conducted for relevant articles published between 2008 and October 2018 in accordance with the PRISMA statement (Moher et al., 2009). Our search terms comprised three blocks; the first search block included keywords relating to depression in older populations. The second search block contained keywords for neuroimaging methods, MRI in particular. To reduce the number of irrelevant hits, a third search block was added, which contained keywords related to specific brain regions and two main networks: the executive control network (ECN) and default mode network (DMN), subserved by the frontal-executive and corticolimbic circuits respectively. In addition, the reference lists of previous systematic reviews and meta-analyses were scanned for articles of interest. The search criteria were conducted in three electronic databases: the Medline/PubMed (Table 1), PsycInfo, and EMBASE. The search strategy was reviewed and approved by a librarian at the Center for Addiction and Mental Health, after modifying the search terms for each database (Figure 1).

**Table 1:** Search blocks: MeSH terms adapted for Medline.

**Geriatric depression concept**	**Neuroimaging concept**	**Neural/Circuitry concept**
1. exp Depressive Disorder 2. exp Depression/ 3. [(late-life or late-onset or “late in life” or geriatric) adj3 depress*].mp. 4. or/1–3	5. Neuroimaging/ or diffusion tensor imaging/ or exp functional neuroimaging/ 6. Magnetic resonance imaging/ or diffusion magnetic resonance imaging/ or echo-planar imaging/ 7. or/5–6	8. Cognitive reserve/ or executive function/ 9. Frontal lobe/ or prefrontal cortex/ or exp hippocampus/ 10. Neural pathways/ or internal capsule/ or perforant pathway/ 11. Default mode network*.mp. 12. (Executive control or executive function* or cognitive control or Corticolimbic or frontal-executive or neural pathway* or default mode network*).mp. 13. or/8–12

### Screening Procedure

After excluding duplicates, all articles identified by the search were screened based on their title or abstract when needed (D.M.) to identify studies using MRI in individuals with LLD. Twenty percent of all articles were additionally screened by a second rater (N.R.R.) to assess the consistency of screening. Then, all retained articles were screened again (N.R.R.), based on their abstracts or full text when needed, to identify studies meeting the eligibility criteria.

### Inclusion and Exclusion Criteria

A study was included if: (1) the study was published in an English language peer-reviewed journal; (2) the study individuals were 55 years or older; (3) the study sample included older individuals diagnosed with a major depressive disorder by a psychiatrist according to DSM or ICD criteria (the depressive episode could be current or in remission); (4) the study included a group of “healthy controls” (HC) with no psychiatric diagnosis; (5) DWI or rs-fMRI was collected and analyzed for both LLD and HC individuals; (6) the results were presented specifically in terms of involved or uninvolved brain circuits. We did not include studies focused exclusively on MCI or dementia but we also included studies that, in addition to individuals with LLD and HC, included a group with MCI or LLD+ MCI, since these studies provided a rare opportunity for “head-to-head” comparison of brain structure and function and further exploration of our hypotheses. We excluded studies that were focused on (1) depressive symptoms exclusively; (2) apathy; and (3) clinical trials (if the baseline/prior to treatment data was available, data were included).

#### Data Extraction/Abstraction

Data from manuscripts meeting the inclusion criteria were extracted and entered into a database that included authors, year of publication, age group, study type, gender composition, sample size, depression status (active or remitted), diagnostic criteria or instrument used to assess depression, presence of other groups (i.e., MCI or LDD+MCI), neuroimaging modality, image analysis methods, and a brief description of findings. One study was excluded at this stage, as we detected potential plagiarism, which was reported to the editor-in-chief of both the original journal (Yin et al., 2016b) and the second journal in which the same data were published by different authors (Zhu et al., 2018).

#### Quality Assessment

A modified version of the Newcastle-Ottawa Scale (NOS) (Table 2) was used to assess the quality of all studies included in the data-synthesis (Wells et al., 2016). The question on representativeness of cases was removed as all cases were consecutive. The scale was modified to check the adequacy of sample size per group, and *n* = 30 was set as the minimum required number per group (Pajula and Tohka, 2016). Since cognitive deficits are important in the link between depression and ADRD, one question was added to assess the quality of cognitive assessment in the studies. In addition, an extra point was given to studies that had used a comprehensive battery to evaluate cognitive performance in both cases and controls. Points were summed and ranged from 0 to 8; and the overall quality of the studies was categorized into weak ( ≤ 3), fair (4–5), or good (6 ≤ ). Among 37 studies included in the systematic review, 20 were categorized as good quality, 15 were fair quality, and 2 were low quality.

**Table 2:** Modified Newcastle-Ottawa scale.

**Modified Newcastle-Ottawa scale**	
	**Ascertainment of diagnosis (LLD, MCI, LLD+MCI)**
1	Independent validation (1+ person or process) to ensure diagnostic accuracy using DSM-IV or 5
0	No independent validation process
0	No description
	**Control definition**
1	Explicitly states that controls have no history of mental illness
0	Not specified
	**Selection of controls**
1	Community controls
0	Selected from a specific population (e.g. hospitals)
0	No description
	**Sample size is adequate**
1	Minimum of 30 per group
0	Less than 30 per group
	**Comparability of groups on the basis of the design or analysis (LLD, MCI, MCI+LLD, HC)**
1	Groups are matched for age or gender and/or analyses are adjusted for age or gender
0	No description of comparability based on factors of interest
	**Assessment of cognitive state in patients and controls**
1	General cognitive tests were used (MMSE, CDR)
1	Comprehensive cognitive tests were used (additional point)
0	No assessment reported
	**Correction for multiple comparisons**
1	Clear description of process to correct for multiple comparisons in analyses
0	No description of process to correct for multiple comparisons in analyses
8	6+/8 good, 4+/8 moderate, 3-/8 poor

Our quality assessment revealed that 32.4% of included studies had only used MMSE for cognitive screening that lacks sensitivity to subtle changes in cognition and has been shown to be influenced by ceiling and floor effects (Tombaugh and McIntyre, 1992). As a result, the confounding effect of cognitive impairment and LLD was indistinguishable in these studies. The Newcastle-Ottawa Scale for each study is presented in Supplementary Tables 1, 2.

## Results

### Selected Studies

Our search identified a total of 4,104 references in the three databases (Figure 1). After removal of duplicates, 3,962 unique citations remained. After screening by title 3,854 were excluded because they focused on psychiatric disorders other than LLD. All remaining citations were screened by their abstract and 33 studies met our eligibility criteria (i.e., the excluded studies did not use DWI or rs-fMRI, reported solely treatment response, did not include a healthy control group, or were conference presentations). As per above, one study was excluded at this stage, as we detected potential plagiarism. Finally, five studies were obtained by cross-reference of previous systematic reviews and meta-analyses and were incorporated into the final pool. Finally, 37 studies met all of our eligibility criteria:15 used DWI; 18 used rs-fMRI; and 4 used both DWI and rs-fMRI (Sexton et al., 2012a; Tadayonnejad et al., 2014; Yin et al., 2016a; Harada et al., 2018). Seven studies included groups other than LLD and controls: three included individuals with MCI (Bai et al., 2012; Xie et al., 2013; Chen et al., 2016); one included individuals with LLD plus amnestic-MCI (LLD+aMCI) (Li et al., 2015b); one included individuals with LLD plus memory deficit (LLD+MD) (Mai et al., 2017); and two included both individuals with aMCI, and LLD plus aMCI (Li et al., 2014, 2015a). Overall, the 37 studies included 1,140 individuals with LLD; 1,211 healthy controls; 173 aMCI; 53 LLD+aMCI; and 15 LLD+ MD. The results are presented below based on the imaging modality (DWI or rs-fMRI).

### DWI Studies

Of 17 DWI studies, five used the ROI analysis method, three used tractography, nine used Tract-Based Spatial Statistics (TBSS), four used network and graph theory analyses, and one study compared apparent fiber density (AFD) and thickness of a single ROI (corpus callosum) in the LLD and HC groups (Table 3). Three DWI studies included additional comparison groups (MCI or LLD+MCI) (Table 4).

**Table 3:** DWI studies with LLD and HC only.

**References**	**N** **(LLD/HC)**	**Study type**	**Depression status**	**Analysis method**	**Frontal-executive findings**	**Corticolimbic findings**	**Other findings**
					**LLD vs. HC**	**LLD vs. HC**	**LLD vs. HC**
Emsell et al. (2017)	107 (55/52)	Cross-sectional	Current	ROI (FA, AFD, thickness) CC^1^	Lower AFD, No difference FA or thickness in CC^1^	–	–
Yuan et al. (2010)	70 (37/33)	Cross-sectional	Remitted LOD>60	ROI (FA) gCC^2^, sCC^3^, SLF^4^, CB^5^, IFOF^6^	Lower FA in gCC No difference FA in sCC, SLF	Lower FA in posterior cingulum No difference FA in middle cingulate bundles	Lower FA in IFOF
Shimony et al. (2010)	96 (73/23)	Cross-sectional	Current	ROI (MD, RA)	Higher MD and lower RA in superior, middle, inferior frontal, medial and lateral orbital frontal ROIs Lower RA in temporal, parietal ROIs	Higher MD in dorsal, anterior, ventral cingulate	Lower RA in occipital, and motor ROIs
Harada et al. (2016)	106 (45/61)	Cross-sectional	Current	TBSS (FA, MD)	–	–	NS
				ROI (FA) UNC^7^	–	Lower FA in left UNC	No WMH difference
Mettenburg et al. (2012)	67 (51/16)	Cross-sectional	22 Remitted, 29 non-remitted	TBSS (FA, MD, AD, RD)	Corrected Voxel-wise comparison Sig clusters of lower FA and Greater extent of higher MD within CC^1^ Higher RD in CC^1^, and bilateral subcortical parietal/temporal white matter	Sig clusters of lower FA and greater extent of higher MD in confluent sections of the CB^5^, Higher RD in CB^5^, UNC^7^ and right Pcun^9^ regions	**Remitted vs. non-remitted** NS
				ROI (FA, MD, AD, RD) CB^5^, gCC^2^, bCC^11^, sCC^3^, and UNC^7^	**Non-remitted vs. HC** Higher RD in gCC^2^, sCC^3^, bCC^10^, Lower FA only in sCC^3^ **Remitted vs. HC** A similar trend, but did not reach significance	**Non-remitted vs. HC** Lower FA, higher RD in right uncinate region **Remitted vs. HC** Higher RD in right UNC	**Remitted and non-remitted vs. HC** No difference FA in gCC, bCC
Sexton et al. (2012a,b)^a^^,^ ^b^	61 (36/25)	Cross-sectional	Current or remitted	TBSS (FA, RD, AD)	–	–	Widespread lower FA in 36% of skeleton voxels at *P* <0.05 and 16% at *P* <0.01 38% percent of voxels with lower in FA had higher DR, but no difference in DA
				ROI (FA, RD, AD) gCC^2^, bCC^10^, sCC^3^, SLF^4^, CB^5^, UNC^7^, ATR^11^, ILF^12^, CST^13^, fornix	Lower FA at *uncorrected* threshold in ATR^11^, sCC^3^, SLF^4^ (more than 50% of voxels at *p* <0.05)	Lower FA at *uncorrected* threshold in UNC^7^ (more than 50% of voxels at *p* <0.05)	Lower FA at *uncorrected* threshold in CST^13^ (more than 50% of voxels at *p* <0.05) No WMH^8^ differences
Alves et al. (2012)	35 (17/18)	Cross-sectional	Current	TBSS (FA, MD)	–	Lower FA at *correcte*d threshold In right PCC^14^	
Guo et al. (2014)	30 (15/15)	Cross-sectional	Current	TBSS (FA)		Lower FA at *corrected* threshold in PGH^15^	
Bezerra et al. (2012)	83 (47/36)	Cross-sectional	Current	TBSS (FA)			No significant difference in FA or MD
Tadayonnejad et al. (2014)^a^	25 (10/15)	Cross-sectional	Current	TBSS (FA, MD)	20% reduction in FA only at *uncorrected* threshold In right forceps minor of the CC^1^		
Colloby et al. (2011)	68 (38/30)	Cross-sectional	Current or remitted	TBSS (FA, MD)	Lower FA only at *uncorrected* threshold in middle temporal	lower FA only at *uncorrected* threshold in PGH^9^	lower FA only at *uncorrected* threshold in fusiform gyri
Harada et al. (2018)^a^	46 (16/30)	Cross-sectional	Current and remitted (same individuals)	Probabilistic tractography between the ACC^16^ and pSTG^17^ sphere	**Current and remitted LLD vs. HC** higher MD, RD, AD, but no difference in FA in the left ACC–pSTG **Current LLD vs. remitted LLD** No difference		
Yin et al. (2016a)^a^	71 (32^c^/39)	Cross-sectional	Current (no antidepressant therapy 6 months prior to the study)	Tractography- deterministic for DMN^19^ [+PCC^14^/Pcu^9^ seed FC]	lower FA and higher RD in tracts connecting PCC^14^/ Pcu^9^ with dACC^18^ No difference FA in tracts connecting PCC^14^/Pcu^9^ with the thalamus		-
Charlton et al. (2014)	46 (23/23)	Cross-sectional	Current	Tractography - deterministic (FA, MD, AD, RD) Bilateral UNC^5^ and cingulum tracts	-	Loss of white matter integrity in the right UNC^5^ (FA, MD, AD, RD), left cingulum (MD, RD) and right cingulum (MD, AD, RD)	-
Li X. et al. (2017)	48 (24/24) **1 year follow-up** 29 (10/ 19)	Cohort 1-year follow-up	Remitted	Between-hemisphere connectivity and graph theory analysis (Probabilistic tractography- AAL ROIs)	**LLD hub regions** **(but not in HC)** L-ITG^20^, and L- MTG^21^	–	**LLD hub regions** left calcarine fissure and surrounding cortex **HC hub regions** R-SMA^22^ **LLD vs. HC** Significantly lower between-hemisphere connectivity, global efficiency, global circuitry strength Sig higher shortest path length **1-year follow up LLD** Lower global efficiency and strength and increased shortest path length
Charlton et al. (2015)	76 (28/48)	Cross-sectional	Current (2 weeks medication free)	Graph theory	**LLD hub regions** Stronger prefrontal region **HC hub regions** stronger right temporal regions	–	No significant differences on global metrics

a *studies that had reported findings in both rs-fMRI and DWI*.

b *DWI findings were reported in two separate publications*.

c participants were over 55 y/o

1 corpus callosum (CC);

2 genu of corpus callosum (gCC);

3 splenium of corpus callosum (sCC);

4 superior longitudinal fasciculus (SLF);

5 cingulate bundle (CB);

6 inferior fronto-occipital fascicle (IFOF);

7 uncinate fasciculus (UNC);

8 White-Matter Hyperintensity (WMH);

9 precuneus (Pcun);

10 body of corpus callosum (bCC);

11 anterior thalamic radiation (ATR);

12 inferior longitudinal fasciculus (ILF);

13 corticospinal tract (CST);

14 posterior cingulate cortex(PCC);

15 parahippocampal gyrus (PGH);

16 anterior cingulate cortex (ACC);

17 posterior superior temporal gyrus (pSTG);

18 dorsal anterior cingulate cortex (dACC);

19 default mode network(DMN);

20 inferior temporal gyrus(ITG);

21 middle temporal gyrus(MTG);

22 *supplementary motor area (SMA)*.

**Table 4:** DWI studies with LLD, HC, and additional comparison groups.

**References**	**N** **(LLD/HC/MCI/** **LLD+aMCI)**	**Study type**	**Depression status**	**Analysis method**	**Frontal-executive findings**	**Corticolimbic** **findings**	**Other findings**
Li et al. (2014)	84 (20/33/18/13)	Cross-sectional	Current	ROI (FA, MD, AD, RD) CCG^1^, CGH^2^, CC^3^, fornix, UNC^4^	Corrected *p* <0.05 **LLD vs. HC** Higher MD, AD, RD in fornix higher AD in CC^3^ **LLD+aMCI vs. HC** Higher MD, AD and RD in fornix	**LLD vs. HC** higher MD, AD, RD in CGH^2^, higher AD in UNC^4^ tracts **aMCI vs. HC** higher MD, RD in CGH^2^ **LLD+aMCI vs. HC** higher MD, AD, RD in CGH^2^	
				Tract-specific voxelwise in the five ROIs	**LLD vs. HC** Higher FA in CC tracts Higher MD in fornix tracts **LLD vs. aMCI** Higher MD in CC^3^ tracts **LLD vs. LLD+aMCI** Lower FA in CC^3^ tracts **aMCI vs. HC** Lower FA, higher MD in fornix tracts **LLD+aMCI vs. HC** Lower FA, higher MD in fornix tracts **LLD+aMCI vs. aMCI** Lower FA, higher MD in CC^3^ tracts Higher MD in fornix tracts	**LLD vs. HC** Lower FA in UNC^4^ tracts Increased MD CGH^2^ **LLD vs. aMCI** Lower MD in CGH and UNC^4^ **LLD vs. LLD+aMCI** Higher MD in UNC, CCG^1^ **aMCI vs. HC** Lower FA in UNC^4^ Lower FA, higher MD in CGH^2^ **LLD+aMCI vs. HC** Lower FA, higher MD in UNC^4^ Higher MD CGH^2^ **LLD vs. aMCI** Lower MD in CGH and UNC **LLD vs. LLD+aMCI** Higher MD in UNC^4^, and CCG^1^ **LLD+aMCI vs. aMCI** Lower FA in UNC tracts **LLD+aMCI vs. all other** Lower FA in bilat-CGH^2^	
Mai et al. (2017)	69 (24/30/-/15^a^)	Cross-sectional	Current	Network analysis probabilistic tractography (AAL atlas regions)	**LLD+MD vs. LLD** Decreased connections nodes: Frontal (R-IFG-operc^5^, R-L-IFG- triang^6^, L-inf-ORB^7^, R-mid-TPO^8^), subcortical (R-L-Put^9^, R-L-Thal^10^), and temporal (L-MTG^11^)	**LLD+MD vs. LLD** Decreased connections nodes: paralimbic (R-med-SFG^12^, R-sup.med-ORB^13^, R-ACC^14^, L-MCC^15^, R-Pcun^16^), subcortical (R-L-Hippo^17^)	**LLD vs. HC** Stronger local connection, lower circuitry density **LLD vs. LLD+MD** Stronger local connection **LLD and LLD+MD vs. HC** Weaker rich-club connection **LLD vs. LLD+MD** Stronger local connection **LLD+MD vs. LLD and HC** Weaker connective strength, lower shortest path length, global efficiency, fault tolerant efficiency
Bai et al. (2012)	103 (35/30/38/-)	Cross-sectional	Remitted	Graph theory	**LLD and aMCI vs. HC** Lower nodal efficiency in frontal and parietal cortices	**LLD hub region** Left Pcun^16^ **LLD vs. aMCI** Higher nodal efficiency in PCC^18^	**LLD vs. HC** Lower circuitry strength global efficiency, higher absolute path length LLD vs. aMCI: no difference

a *memory deficit LLD (LLD+MD)*.

1 Cingulum-cingulate gyrus tract (CCG);

2 Cingulum-hippocampus tract (CGH);

3 corpus callosum (CC);

4 uncinate fasciculus (UNC);

5 opercular part of the inferior frontal gyrus (IFG-operc);

6 triangle part of the inferior frontal gyrus (IFG- triang);

7 orbital part of the inferior frontal gyrus (inf-ORB);

8 temporal pole of middle temporal gyrus (mid-TPO);

9 putamen (Put);

10 thalamus (Thal);

11 middle temporal gyrus (MTG);

12 medial part of the superior frontal (med-SFG);

13 medial orbital part of the superior frontal gyrus (med-sup-ORB);

14 anterior cingulate cortex (ACC);

15 middle cingulate cortex (MCC);

16 precuneus (Pcun);

17 hippocampus (Hippo);

18 *posterior cingulate cortex (PCC)*.

#### DWI Studies Using ROI Analysis

Seven studies compared white matter integrity using ROI analysis of which six compared differences between individuals with LLD and an HC group, and one across individuals with LLD, aMCI, and LLD+aMCI, and an HC group (Li et al., 2014). Additionally, three studies conducted TBSS (Mettenburg et al., 2012; Sexton et al., 2012a; Harada et al., 2016), and tract-specific voxelwise analysis (Li et al., 2014) presented in the section DWI Studies Using TBSS.

Individuals with LLD vs. HC groups, showed disruption in white matter integrity in frontal-executive (Shimony et al., 2010; Yuan et al., 2010), and corticolimbic tracts (Shimony et al., 2010; Yuan et al., 2010; Harada et al., 2016). However, these studies have also found no significant difference between individuals with LLD and an HC group in other frontal-executive (Shimony et al., 2010; Yuan et al., 2010; Sexton et al., 2012a) and corticolimbic tracts (Yuan et al., 2010; Sexton et al., 2012a). One study reported no difference in the FA values or thickness, but a lower AFD in the corpus callosum [frontal-executive] (Emsell et al., 2017). See Table 3 for further details.

One other study compared individuals with non-remitting and remitting LLD and an HC group and found lower FA in the splenium of the corpus callosum (frontal-executive) and the cingulate bundles and higher RD in the right uncinate fasciculus (corticolimbic) in individuals with non-remitting depression (but not in individuals with remitting depression) compared to the HC group (Mettenburg et al., 2012) (Table 3).

In the study comparing white matter integrity across LLD, aMCI, LLD+aMCI, and HC groups, individuals with LLD demonstrated higher MD and/or AD in the fornix, the corpus callosum (frontal-executive), the cingulum-hippocampus part, the uncinate fasciculus (corticolimbic) vs. the HC group. However, there was no significant difference between individuals with LLD and aMCI or LLD+aMCI (Li et al., 2014). See Table 4 for further details.

#### DWI Studies Using TBSS

Nine studies used TBSS analyses of white matter, of which eight compared individuals with LLD to an HC group and one across four groups (LLD, HC, aMCI, LLD+aMCI). Two studies found no significant difference in FA or MD value (Bezerra et al., 2012; Harada et al., 2016); and three reported lower FA only at uncorrected threshold (Colloby et al., 2011; Sexton et al., 2012a; Tadayonnejad et al., 2014). Four studies reported loss of white matter integrity at corrected threshold in individuals with LLD vs. the HC group. In these studies, the loss of white matter integrity was found in both the frontal executive tracts (i.e., fornix, corpus callosum) (Mettenburg et al., 2012; Li et al., 2014), and the corticolimbic tracts (i.e., cingulate bundles, uncinate fasciculus, posterior cingulate gyrus, parahippocampal gyrus, uncinate fasciculus) (Alves et al., 2012; Mettenburg et al., 2012; Guo et al., 2014; Li et al., 2014). In the tract-specific voxelwise analysis of the selected ROIs, individuals with aMCI and LLD+aMCI demonstrated similar findings compared to the HC group (Li et al., 2014). See Tables 3, 4 for further details.

#### DWI Studies Using Tractography

Three DWI studies used tractography to compare white matter integrity in individuals with LLD to a HC group. Two studies connected one or two corticolimbic [posterior cingulate cortex/precuneus (PCC/Pcun), left anterior cingulate cortex (l-ACC)] regions to one or two frontal-executive [dorsal anterior cingulate cortex (dACC), thalamus, posterior superior temporal gyrus (pSTG)] regions. In the first study, individuals with LLD showed a lower FA and higher radial diffusivity (RD) in the tracts connecting the PCC/Pcun with the dACC, but no differences in the tracts connecting the PCC/PCun with the thalamus (Yin et al., 2016a). In the second study, both individuals with current and remitted LLD had a higher MD, RD, AD but no difference in FA compared to the HC group in the tract connecting l-ACC to pSTG (Harada et al., 2018). Finally, a corticolimbic tract study connecting bilateral uncinate fasciculus tracts and bilateral cingulum tracts, revealed a loss of white matter integrity in the right uncinate fasciculus and bilateral cingulum tracts in individuals with LLD (Charlton et al., 2014). See Table 3 for further details.

#### DWI Studies Using Network and Graph Theory Analysis

Four DWI studies used network analysis (Li X. et al., 2017; Mai et al., 2017) and graph theory analysis methods (Bai et al., 2012; Charlton et al., 2015). In these four studies, T1-weighted images were used either as seed regions for probabilistic tractography or to generate label maps after tracking the whole brain at each voxel. Global efficiency is simply the average of the efficiencies over all pairs of vertices (node connections). Clustering coefficient is the overall level of clustering in a network, i.e., the average degree to which nodes in a graph tend to cluster together. Network strength is a measure of the connectivity of a graph, where a greater value means more connection strength from one node to its neighbor nodes. The average path length is the average number of steps along the shortest paths for all possible pairs of network nodes (Tables 3, 4).

##### Global metrics

Two studies compared LLD individuals with current depression to a HC group, and reported either no difference in the global metrics (Charlton et al., 2015), or weaker rich-club connection, decreased shortest path length, stronger local connection and lower network density in individuals with LLD (Mai et al., 2017). Two other studies compared individuals with remitted LLD to a HC group and found reduced global efficiency and global strength in individuals with LLD (Bai et al., 2012; Li W. et al., 2017). In addition, similar disruption of the global metrics (i.e., lower global efficiency, weaker connection strength, lower shortest path length) was found in the LLD+memory deficit (LLD+MD) (Mai et al., 2017), and individuals with aMCI compared to the HC group (Bai et al., 2012). However, individuals with LLD had a stronger local connection compared to individuals with LLD+ memory deficit (Mai et al., 2017), and showed no difference compared to individuals with aMCI (Bai et al., 2012).

##### Hub regions and nodal efficiency

In addition to global metrics differences, the studies reviewed here reported alteration in the hub distribution and nodal efficiency in individuals with LLD compared to the HC group. For instance, a reduction of nodal efficiency in frontal and parietal cortices was found in the remitted LLD group (Bai et al., 2012), however LLD individuals with current depression showed stronger prefrontal region hubs (Charlton et al., 2015). Furthermore, regions from both frontal-executive (i.e., Inferior and middle temporal gyri) and corticolimbic circuits (i.e., precuneus) were identified as hub regions in the individuals with LLD but not in the HC group (Bai et al., 2012; Li W. et al., 2017).

In the comparison between individuals with LLD and aMCI, those with LLD had a higher nodal efficiency in the posterior cingulate (PCC) (Bai et al., 2012). In the study by Mai et al. (2017) individuals with LLD compared to LLD+MD showed increased nodal connection in the frontal, paralimbic, subcortical, and some parietal and temporal regions, which belong to the frontal-executive and corticolimbic circuits.

### rs-fMRI Studies

We included 22 rs-fMRI studies; 10 used seed-based functional connectivity (FC); two used ReHo (regional homogeneity); one used both seed-based FC and ReHo, two used ICA; two used graph theory analysis; and five used other methods (Table 5). Four studies had additional comparison groups included in their analysis (Table 6). In this review, we focused on two main functional networks associated with frontal-executive and corticolimbic circuit, the executive control network (ECN) and the default mode network (DMN).

**Table 5:** rs-fMRI studies with LLD and HC only.

**References**	**N** **(LLD/HC)**	**Study type**	**Depression status**	**Analysis method**	**Frontal-executive findings** **LLD vs. HC**	**Corticolimbic findings** **LLD vs. HC**	**Other findings** **LLD vs. HC**
Ikuta et al. (2017)	95 (33/62)	Cross-sectional	Current	Seed dorsal raphe nucleus (DRN)	–	Lower FC between DRN and bilat. PCC^1^	–
Yin et al. (2015)	71 (32 LOD/39)	cross-sectional	Current (no antidepressant therapy 6 months prior to study	Seed (Cerebellum)	Decreased FC in left dlPFC^2^, and bilateral cerebellum	Increased FC in vmPFC^3^ and ACC^4^ Decreased FC in PCC^1^	Increased FC in SMA^5^ and bilat. SMG^6^
Alalade et al. (2011)	29 (11/18)	Cross-sectional	Current	Seed (Cerebellum)	Decreased FC 1. R-Crus II_Exec_ with R-dlPFC^2^ 3. R-Crus I_DMN_ with R-head of the caudate, R-insula/putamen, 4. VI_Limbic_ with R-IPC^7^ 5. L-Vermis_Limbic_ with L-vlPFC^8^	Decreased FC 1. R-Crus II_Exec_ seed R- dmPFC^9^ 2. bilal-Crus I_Exec1_ R-Crus II_Exec2_, and left Vermis_Limbic_ with vmPFC^3^ 3. VI_Limbic_ with PCC^1^	Decreased FC between left 1. Lobule V_Motor_ with L-dlPFC^2^ and vlPFC^8^ 2. R-Crus I_DMN_ and L-FFG^10^
Alexopoulos et al. (2012)	26 (16/10)	12-week clinical-trial. (Only baseline findings were extracted).	Current	Seed (ACC^4^, dlPFC^2^)	Decreased FC seed L-dACC^11^ with L-dlPFC seed L-dlPFC^2^ with bilat. IPC^7^	–	–
				Seed (PCC^1^/Pcun^8^)	Increased FC in lateral parietal regions and L-Pcun^12^	Increased FC in DMN andsACC^13^, and vmPFC^3^	–
Yin et al. (2016a)^a^	71 (32/39)	Cross-sectional	Current (no antidepressant therapy 6 months prior to the study)	Seed (PCC^1^/Pcun^8^) for DMN^14^	Decreased FC with and dACC^11^ and thalamus	Both LLD and HC showed typical distribution of the DMN	–
Wu et al. (2011)	24 (12/12)	12-week clinical-trial. Only baseline findings were extracted.	Current	Seed (PCC^1^)(low cognitive load, event-related task)	Increased FC in the dmPFC^9^ and OFC^15^	Decreased FC in sACC^9^	Higher WMH
Shu et al. (2014)	60 (31/29)	Cross-sectional	Remitted	Seed(Hippo^16^)	**R-hippo positive** Circuit: increased FC to R-pMTG^17^ **R-hippo negative** Circuit: decreased FC to R-SPL^18^	**R-hippo positive** Circuit: decreased FC to L-MFG^18^ **L- hippo positive** Circuit: decreased to L-mPFC^19^ and MFG^18^ **L-hippo negative** Circuit: increased FC to R- MFG/SFG^20^	**R-hippo positive** Circuit: increased FC to LG^21^ and cuneus and decreased FC to SMA^5^ **L-hippo negative** Circuit: decreased FC to R-IOG^22^/LG^21^
					**APOE** **ε4 carrier vs. non-carrier right hippo**: Decreased positive FC to ITG^23^ and R-IFG^24^; and decreased negative FC to L-IPL^25^.	**APOE** **ε4 carrier vs. non-carrier** **left hippo:** Decreased positive FC to bilat. mPFC^19^/ACC^4^ regions **right hippo**: Decreased positive FC to bilat. mPFC	**APOE** **ε4 carrier vs. non-carrier** **left hippo**: Decreased negative FC to R-SMG^26^; and increased positive FC to bilat. insula. **right hippo**: Increased positive FC to L-Insula
Wang et al. (2015)	30 (14/16)	Cohort - 21 months follow up	Remitted	Seed (6 Hippo subregions)	**Both baseline and follow-up vs. HC** All Hippo sub-regional seeds showed lower and less diffuse FC with parietal cortex **From baseline to follow-up** Nearly all Hippo subregions showed increased FC mainly with mainly frontal cortex	**From baseline to follow-up** Greater decrease in the left CA^27^ FC with the bilat PCC^1^/Pcun^12^	–
Yue et al. (2013)	44 (22/22)	Cross-sectional	Current (first onset medication-naïve)	Seed amygdala (Amy)	L- Amy positive circuit: decreased FC with R-MFG^18^	L- Amy positive circuit: reduction FC with L-SFG^20^	L- Amy negative circuit: increased FC in R-postCG^28^ R-Amy negative circuit: reduction FC in R-MOG^29^
				ReHo maps	Decreased ReHo in R-MFG^18^	decreased ReHo in L-SFG^20^	–
Chen et al. (2012)	30 (15^b^/15)	Cross-sectional	Current, treatment-naïve	ReHo maps	Increased ReHo in L-STG^30^, L- Crus I cerebellum	Deceased ReHo in R-Pcun^12^	–
Yuan et al. (2008)	32 (18/14)	Cross-sectional	Remitted^c^	ReHo maps	Deceased ReHo in L-MFG^18^, R-STG^30^ and MTG^31^ Increased ReHo in bilat-putamen	Deceased ReHo in bilat SFG^20^ and bilat PcunIncreased ReHo in R-SFG^20^	Deceased ReHo in R- FFG^10^ and R-postCG^28^ Increased ReHo in L- postCG^28^
Li W. et al. (2017)	68 (39/29)^d^	Cross-sectional	Current	Voxelwise ICA (ECN^32^, DMN, SN^33^)	**Intra-circuit intrinsic FC comparisons** **ECN**: - Increased positive FC in bilat. IPL^25^ - Decreased negative FC in bilat insula, sACC^13^, and MCC^33^ - Decreased negative FC and reversal FC pattern to positive in bilat-PCC^1^, L-MTL^34^, PHG^35^, Hippo, Amy **DMN**: - Decreased positive FC in R-SFG^24^ - Decreased negative FC in L-Insula and R-SPL^18^ - Increased negative FC in L-PHG^35^ **SN**: - Decreased positive FC in R-dlPFC^2^ **Intrinsic circuit connectivity matrix**: 1. Decreased positive inter-circuit FC between bilat-ECN and subcortical-DMN 2. Decreased negative FC between L-ECN and SN (insula). 3. Increased inter-circuit FC between L-ECN and post-DMN + reversal from negative to positive FC 4. Increased positive FC between L-ECN and dACC^11^		
Sexton et al. (2012a)	61 (36/25)	Cross-sectional	Current or remitted	dual regression ICA	NS in ECN	NS in DMN	–
Harada et al. (2018)^a^	46 (16/30)	Cross-sectional	Current and remitted (same individuals)	FC ACC ~ pSTG^36^	**current LLD vs. HC** NS **remitted LDD vs. HC** decreased FC in L-ACC–pSTG **current LLD vs. remitted LLD** NS		–
Yue et al. (2015)	32 (16/16)^e^	Cross-sectional	Current	ALFF	**Effect of disease:** ALF widely distributed over CePL^37^, CeAL^38^, and R-SPL^18^	**Effect of disease:** ALFF widely distributed over L-middle-OFC Effect of disease*frequency: distributed over R-SFG^20^	**Effect of disease:** ALFF widely distributed over L-SOG^39^
Hou et al. (2016)	68 (31/37)^f^	Cross-sectional	Current	Voxel-mirrored homotopic connectivity (VMHC)	Lower VMHC in STG^30^, CePL^37^	Lower VMHC in SFG^20^	Lower VMHC in postCG^28^ and preCG^40^
Tadayonnejad et al. (2014)^a^	25 (10/15)	Cross-sectional	Current	Pairwise BOLD signal averages correlations after Fisher's r-to-z transformations	Lower FC between R-Accumb^41^ and R-mOFC^42^)	Lower FC between R-rACC^43^ and bilat-SFG^20^	–
Yin et al. (2016b)	64 (33/31)^g^	Cross-sectional	Remitted	Graph theory of DMN	–	Decreased FC in DMN	Abnormal global topology increased characteristic path length and reduced global efficiency of DMN

a *Studies that had reported findings in both rs-fMRI and DWI*.

b *Illness duration was less than 1 year, and individuals were treatment-naive. In the current review we excluded results of 15 young EOD and 15 young HCs (mean age 24)*.

c *Individuals were in the first depressive episode and the age of onset was over 60 years, and remitted for more than 6 months before the enrollment*.

d *Did not exclude significant anxiety or mild cognitive impairment as long as the primary diagnosis was LLD*.

e *Individuals with first onset after 60 years and medication-naïve*.

f *First depressive episode and the age of onset was over 55 years, Pearson's correlation analysis was conducted*.

Between each pair of time series within symmetrical interhemispheric voxels. The computed correlation coefficients were Fisher z-transformed to obtain a VMHC z-map for statistical analyses.

g *Duration of illness was less than 5 years and a medication-free period for all individuals was longer than 3 months prior to the assessment, GT threshold: 0.10 to 0.40 using an increment of 0.01*.

1 Posterior cingulate cortex (PCC);

2 dorsolateral prefrontal cortex (dlPFC);

3 ventromedial prefrontal cortex (vmPFC);

4 anterior cingulate cortex (ACC);

5 supplementary motor area (SMA);

6 supramarginal gyrus (SMG);

7 Inferior parietal cortex (IPC);

8 ventrolateral prefrontal cortex (vlPFC);

9 dorsomedial prefrontal cortex (dmPFC);

10 fusiform gyrus (FFG);

11 dorsal anterior cingulate cortex (dACC);

12 precuneus (Pcun);

13 subgenual anterior cingulate cortex (sACC);

14 default mode network (DMN);

15 orbitofrontal cortex (OFC);

16 hippocampus (Hippo);

17 posterior middle temporal gyrus (pMTG);

18 superior parietal lobule (SPL);

19 medial prefrontal cortex (mPFC);

20 superior frontal gyrus (SFG);

21 lingual gyrus (LG);

22 inferior occipital gyrus (IOG)**;**

23 inferior temporal gyrus (ITG);

24 inferior frontal gyrus (IFG);

25 inferior parietal lobule (IPL);

26 middle frontal gyrus (MFG);

27 cornu ammonis (CA);

28 post central gyrus (postCG);

29 middle occipital gyrus (MOG);

30 superior temporal gyrus (STG);

31 executive control network (ECN);

32 salient network (SN);

33 middle cingulate cortex (MCC);

34 medial temporal lobe (MTL);

35 parahippocampal gyrus (PHG);

36 posterior superior temporal gyrus (pSTG);

37 Posterior lobe of cerebellum (CePL);

38 anterior lobe of cerebellum (CeAL);

39 superior occipital gyrus (SOG);

40 precentral gyrus (preCG);

41 nucleus accumbens (Accumb);

42 medial orbitofrontal cortex (mOFC);

43 *rostral anterior cingulate cortex (rACC)*.

**Table 6:** rs-fMRI studies with LLD, HC, and additional comparison groups.

**References**	**N** **(LLD/HC/MCI/** **LLD+MCI)**	**Study type**	**Depression** **status**	**Analysis** **method**	**Frontal-executive** **findings**	**Corticolimbic** **findings**	**Other** **findings**
Xie et al. (2013)	72 (18/25/17/12	Cross-sectional	Current	Seed (Hippo)	**LLD vs. non-depressed**• **Left hippo**: Decreased anti-correlation R-dlPFC^1^• **Right hippo**: Increased FC in bilat-Thal^2^, and R-Lent^3^, decreased anti-correlated FC in l-dlPFC and d-striatum^4^ **MCI vs. non-CI**• **Left hippo**: Decreased + and – FC in bilat-dlPFC, IPC^5^, L- pMTG^6^; and R-dACC^7^ and SPC^8^.• **Right hippo**: Decreased + and - FC in bilat-aTP^9^ and IPC^5^; L-vlPFC^10^, pMTG; R-ITC^11^. Interactive effect of LLD*MCI• **Right hippo**: Found in bilat-MOG^12^, L-dACC, R-dlPFC/dACC cluster	**LLD vs. non-depressed**• **Left hippo**: Increased FC bilat-PCC^13^, and R-dmPFC^14^• **Right hippo**: Increased FC in L-Hippo **MCI vs. non-CI**• **Left hippo**: Decreased + and – FC in bilat-dmPFC• **Right hippo**: Decreased + and – FC in bilat-aTP^9^, PHG^15^ Interactive effect LLD*MCI• **Right hippo**: Found in vmPFC^16^	**LLD vs. non-depressed**• **Right hippo**: Decreased anti- correlated FC in bilat-MOG^13^ **MCI vs. non-CI:**• **Left hippo**: Decreased + and – FC in bilat-RSC^17^; L-postCG^18^
Li et al. (2015b)	63 (25/26/-/15)	Cross-sectional	Current	Seed (Amygdala)	**LLD vs. HC** Decreased FC in R-SPL^19^, MFG^20^, IFG^21^, ITG^22^ Increased in cerebellar vermis **LLD+aMCI vs. HC** Decreased FC in bilat-SPL, R-IFG, MFG; and MTG^23^ Increased FC in IPL^24^ **LLD+aMCI vs. LLD** Decreased FC in L-MTG^20^	**LLD vs. HC** Increased FC in R-PCC^13^ and TP^25^ **LLD+aMCI vs. HC** Decreased FC in left TP, PHG^15^, hippo^26^, cuneus **LLD+aMCI vs. LLD** Greater decrease of FC in the bilateral TP, cuneus (posterior DMN^35^)	**LLD+MCI vs. LLD** Decreased FC in OG^27^ **LLD vs. HC** decreased FC in PreCG^28^ **LLD+aMCI vs. HC** Decreased FC in PreCG, bilat-IOG^29^ and MOG^30^ **LLD+aMCI vs. LLD** Decreased FC in bilat-FFG^31^, IOG, MOG
Chen et al. (2016)	256 (55/114/87)	Cross-sectional	Remitted	Correlation between each pair of 36 seeds that represent five major RSNs^32^	**LLD vs. HC:** Reduced FC degree in bilat-ITG^22^ **MCI vs. HC:** Trend toward increased FC degree at all nodes (except for r-ITG^22^)	**LLD vs. HC:** Reduced FC strength in ECN^33^-DMN^35^ *After controlling for age, sex, education and APOE genotype: reduced correlation in ECN-DMN pair in LLD > MCI vs. HC	**LLD vs. HC:** Reduced FC degree in SMA^36^ **LLD vs. HC:** Reduced FC strength in SMN^37^ **aMCI vs. HC:** A trend toward increased FC strength within SAL and SMN **LLD vs. aMCI:** Reduced FC strength within SAL^38^ and SMN
Li et al. (2015a)	79 (23/25/18/13)	Cross-sectional	Current	Graph theory threshold range (0.03–0.5)	**LLD, aMCI, LLD+aMCI vs. HC** Combined ECN/VAN split into two distinct modules: ECN^33^ and VAN^34^	**LLD and aMCI vs. HC** DMN module was split into two smaller modules	**LLD vs. HC** Disrupted FC segregation (decreased local efficiency) **LLD+aMCI** **>** **LLD** **>** **MCI** Greatest mean nodal efficiency **LLD+aMCI vs. aMCI** Decreased local efficiency **LLD+aMCI vs. all other groups** greatest disruptions in integration (diminished global efficiency) **LLD+aMCI** Had most variable modular community followed by LLD

1 dorsolateral prefrontal cortex (dlPFC);

2 thalamus (Thal);

3 lentiform nucleus (Lent);

4 dorsal striatum (caudate and putamen) d-striatum;

5 inferior parietal cortex (IPC);

6 left posterior middle temporal gyrus (pMTG);

7 dorsal anterior cingulate cortex (dACC);

8 superior parietal cortex (SPC);

9 anterior temporal pole (aTP);

10 ventrolateral prefrontal cortex (vlPFC);

11 inferior temporal cortex (ITC);

12 middle occipital gyrus (MOG);

13 posterior cingulate cortex (PCC);

14 dorsomedial prefrontal cortex (dmPFC);

15 parahippocampal gyrus (PHG);

16 ventromedial prefrontal cortex (vmPFC);

17 retro-splenial cortex (RSC);

18 post central gyrus (postCG);

19 superior parietal lobe (SPL);

20 middle frontal gyrus (MFG);

21 inferior frontal gyrus (IFG);

22 inferior temporal gyrus (ITG);

23 middle temporal gyrus (MTG);

24 inferior parietal lobule (IPL);

25 temporal pole (TP);

26 hippocampus (hippo);

27 occipital gyri (OG);

28 pre-central gyrus (PreCG);

29 inferior occipital gyrus (IOG);

30 middle occipital gyrus (MOG);

31 fusiform gyrus (FFG);

32 resting state networks (RSN);

33 executive control network (ECN);

34 ventral attention network (VAN);

35 default mode network (DMN);

36 supplemental motor area (SMA);

37 sensory-motor network (SMN);

38 *salience network (SAL)*.

#### rs-fMRI Studies Using Seed-Based Connectivity

Ten rs-fMRI studies conducted seed-based connectivity analysis, using a variety of ROIs as seed regions. From the frontal-executive circuit, seed regions were the anterior cingulate cortex, dorsolateral prefrontal cortex (Alexopoulos et al., 2012), and cerebellum (Alalade et al., 2011; Yin et al., 2015). From the corticolimbic circuit, seed regions were the posterior cingulate cortex [PCC]/precuneus [Pcun] (Wu et al., 2011; Alexopoulos et al., 2012; Yin et al., 2016a), hippocampus (Xie et al., 2013; Shu et al., 2014; Wang et al., 2015), and amygdala (Yue et al., 2013; Li et al., 2015b). All studies reported projections of the seed region to ECN, DMN, or both networks. One study used dorsal raphe nucleus (DRN) as the seed region and found significantly lower functional connectivity (FC) in the bilateral PCC in the LLD individuals with current depression vs. the HC group (Ikuta et al., 2017) (Table 5).

In the study using anterior cingulate cortex (ACC) and dorsolateral prefrontal cortex (dlPFC) as the seed region, the LLD individuals with current depression showed decreased FC in the frontoparietal and temporal areas. In addition, remitters vs. non-remitters demonstrated greater FC in the dorsal ACC, dlPFC, and parietal cortices (frontal-executive) (Alexopoulos et al., 2012) (Table 5).

In two studies using cerebellum as the seed region, the LLD individuals with current depression vs. the HC group, showed decreased FC in the frontal-executive regions including the dlPFC (Alalade et al., 2011; Yin et al., 2015), caudate, putamen, and parietal cortex, as well as corticolimbic regions including the PCC (Alalade et al., 2011; Yin et al., 2015), dmPFC (Alalade et al., 2011), and increased FC in the ventromedial prefrontal cortex (vmPFC) and ACC (Yin et al., 2015) (Table 5).

In three studies where PCC/Pcun was used as the seed region, the LLD individuals with current depression compared to the HC group showed decreased FC (Yin et al., 2016a) or increased FC (Wu et al., 2011; Alexopoulos et al., 2012) in the frontal-executive regions [i.e., thalamus, dorsomedial prefrontal (dlPFC), dorsal anterior cingulate cortex (dACC)]. In addition, decreased (Wu et al., 2011) and increased FC (Alexopoulos et al., 2012) was found in the corticolimbic regions (i.e., subgenual ACC, ventromedial PFC).

Three studies used hippocampus or subregions of hippocampus as the seed region and compared the functional connectivity (FC) between individuals with LLD and HC group. One study compared FC in the LLD individuals with current depression to non-depressed individuals and found increased FC and decreased anti-correlation FC in the frontal-executive regions [i.e., dorsolateral prefrontal cortex (dlPFC)] and increased FC in the corticolimbic regions [i.e., posterior cingulate cortex, dorsomedial prefrontal cortex (dmPFC)] (Xie et al., 2013). This study included individuals with MCI that showed decreased FC in both frontal-executive [i.e., dlPFC, dorsal anterior cingulate cortex (ACC)], and corticolimbic (i.e., dmPFC) regions relative to individuals with non-cognitive impairment (Xie et al., 2013) (Table 5).

Two other studies compared hippocampal functional connecivity in individuals with remitted LLD to a HC group. Individuals with remitted LLD showed decreased FC in the frontal-executive (parietal cortex) and corticolimbic (i.e., middle frontal gyri) regions (Shu et al., 2014; Wang et al., 2015). In addition, after 21 months of follow up, the remitted LLD had an increased FC in the frontal cortex (frontal-executive), but a greater decline in the midline posterior regions (PCC/Pcu; corticolimbic) (Wang et al., 2015) (Table 5).

The amygdala was used as the seed region in two studies, comparing FC between LLD individuals with current depression and a HC group. Individuals with LLD demonstrated decreased FC in both the frontal-executive regions (i.e., frontal gyri, temporoparietal cortices), and the corticolimbic regions (i.e., superior frontal gyrus) (Yue et al., 2013; Li et al., 2015b). Furthermore, individuals with LLD had increased FC in some corticolimbic regions was found the midline posterior regions (PCC) and temporal pole compared to the HC group (Li et al., 2015b) (Tables 5, 6).

In addition, in the study by Li et al. (2015b) an additional comparison group (LLD+aMCI) was included. The LLD+aMCI demonstrated decreased FC in the temporoparietal, and frontal gyri and an increased FC in the parietal lobe (frontal-executive). Further, LLD+aMCI demonstrated decreased FC in the posterior midline regions (medial temporal lobe and cuneus; corticolimbic) compared to individuals with LLD and HC group (Li et al., 2015b) (Table 5).

#### rs-fMRI Studies Using ReHo (Regional Homogeneity)

Three studies used ReHo, a method that evaluates the similarity or synchronization between the time series of a given voxel and its nearest neighbors (Zang et al., 2004). Results from three studies, comparing ReHo between the LLD and the HC groups, showed ReHo differences in both frontal-executive and corticolimbic circuits. From the frontal-executive circuit, LLD individuals with current depression and individuals with remitted LLD compared to the HC group showed increased ReHo in the superior temporal gyrus (Chen et al., 2012), and putamen (Yuan et al., 2008), and decreased ReHo in the frontal gyrus (Yuan et al., 2008; Yue et al., 2013) and middle temporal gyrus (Yuan et al., 2008), respectively. From the corticolimbic regions, both individuals with current depression and remitted LLD showed lower ReHo in the superior frontal gyrus (Yuan et al., 2008; Yue et al., 2013), and precuneus (Yuan et al., 2008; Chen et al., 2012) compare to the HC group (Table 5).

#### rs-fMRI Studies Using ICA (Independent Component Analysis)

ICA is a data-driven approach that evaluates the whole brain and decomposes it into a set of independent components, each depicted as a functional map (Van Den Heuvel et al., 2009; Wang N. et al., 2012). Two studies used ICA to investigate alterations in both executive control network (ECN) and default mode network (DMN). One study found no significant difference between individuals with remitted and/or current depression LLD and the HC group (Sexton et al., 2012a). In the other study, individuals with LLD showed increased (i.e., inferior parietal) and decreased positive FC in the frontal-executive regions [i.e., dorsolateral prefrontal cortex (dlPFC)], and decreased positive FC in the corticolimbic regions (i.e., superior frontal) (Li W. et al., 2017) (Table 5).

#### rs-fMRI Studies Using Network and Graph Theory Analysis

Two studies used graph theory analysis. The first study compared four groups (LLD, aMCI, and LLD+aMCI, HC) and found alteration in small-worldness (Sigma) of all three patient groups, as well as separation of the ECN and DMN into two distinct modules in individuals with LLD and aMCI compared to HC group. In addition, individuals with LLD+aMCI had the greatest diminished global efficiency, and the most variable modular community structure compared to all other groups, as well as the greatest disruptions in the mean nodal efficiency followed by individuals with LLD and aMCI (Li et al., 2015a) (Table 5).

The other study compared the alteration of resting-state FC and topological organization of DMN in individuals with remitted LLD to an HC group, and found universally decreased FC and global efficiency, and increased characteristic path length of the DMN compared to the HC group (Yin et al., 2016b) (Table 5).

#### Other Methods

Five studies used other methods. One study (Yue et al., 2015) used Amplitude of Low-Frequency Fluctuations (ALFF) that measures spontaneous fluctuations in BOLD-fMRI signal intensity within the frequency range between 0.01 and 0.1 Hz. Thus, it indexes the intensity of low-frequency oscillations in rs-fMRI (Zang et al., 2007). In this study, the primary effect of disease or the interaction of disease and frequency in the LLD individuals with current depression vs. HC group was distributed over the frontal-executive (i.e., cerebellum, superior parietal) and corticolimbic (i.e., middle orbitofrontal, superior frontal gyri) regions (Yue et al., 2015).

Another study used a voxel-mirrored homotopic connectivity (VMHC) method and found a signal drop in the LLD individuals with current depression compared to the HC group, in the frontal-executive (superior temporal gyrus, posterior cerebellar) and corticolimbic (superior frontal gyrus) regions (Hou et al., 2016) (Table 5).

One study used a pairwise BOLD signal average correlation comparison of 87 regions in currently depressed LLD (Tadayonnejad et al., 2014) and found decreased FC in individuals with LLD compared to the HC group in frontal-executive (i.e., nucleus accumbens) and corticolimbic regions (i.e., rostral anterior cingulate cortex, superior frontal, and middle orbitofrontal gyri).

Harada et al. (2018) used a Pearson's correlation coefficient score between anterior cingulate cortex (ACC) and posterior superior temporal gyrus (pSTG). The LLD individuals with current depression showed no difference compared to the HC group, however individuals with remitted LLD showed lower functional connectivity compared to the HC group (Table 5).

Finally, one study measured correlation degree and strength of functional connectivity (FC) between each pair of 36 seeds representing five major resting state networks (RSN) individuals with remitted LLD, aMCI, and HC group. Individuals with remitted LLD demonstrated reduced degree of FC in bilateral inferior temporal cortices (frontal-executive) compared to the HC group. In addition, individuals with remitted LLD and to a lesser extent individuals with aMCI showed reduced correlation in the executive control network (ECN) and default mode network (DMN) (Chen et al., 2016) (Table 6).

## Discussion

### Overview

We reviewed studies measuring structural (DWI) or functional (rs-fMRI) properties of brain circuits in individuals with LDD—both early-onset depression recurring in late life and late-onset depression, as well as remitted and currently depressed—compared to a HC group. The aim of this review was to systematically investigate the evidence for structural and functional disruption in the frontal-executive and corticolimbic circuits that are implicated in dementia. We included 37 studies that met our eligibility criteria (19 DWI studies, 22 rs-fMRI studies, of which 4 studies had both DWI and rs-fMRI). Most studies but not all reported some impairment in both frontal-executive and corticolimbic circuits. We did not find any DWI or rs-fMRI studies directly comparing LLD with ADRD, however, seven DWI or rs-fMRI studies compared structural and functional alterations of brain circuits among LLD, MCI, or LLD+aMCI, and HC. All seven studies reported alterations in structure (Bai et al., 2012; Li et al., 2014; Mai et al., 2017) and function (Xie et al., 2013; Li et al., 2015a,b; Chen et al., 2016) of frontal-executive and corticolimbic circuits in those with LLD, aMCI and LLD+aMCI compared to HC individuals. Moreover, LLD+aMCI and aMCI groups were found to have more disruption in both circuits when compared to LLD (Bai et al., 2012; Li et al., 2014, 2015a,b; Mai et al., 2017). Although, five of these studies had sample sizes of less than 30 per group (Xie et al., 2013; Li et al., 2014, 2015a,b; Mai et al., 2017), these findings suggest that LLD and MCI share impairment of the same brain circuits and represent a possible continuum in disease progression toward ADRD.

### Testing Our Hypothesis: Evidence and New Findings

In 2008, we proposed several models of biological mechanisms linking depression to Alzheimer's disease and related dementia (Butters et al., 2008). These models provided the insight that frontal-executive and corticolimbic circuits were potentially vulnerable in older adults with depression. We aimed to evaluate MR evidence published since 2008, for alteration in structure (DWI) and function (rs-functional MRI) of these two circuits. Despite the variability across reviewed studies with respect to study design, number of individuals, and analytic methods, some consistent results emerged. Impairment of both frontal-executive and corticolimbic circuits was present in individuals with LLD compared to HCs in some but not all studies. Some of these studies suffered from limitations of small sample sizes and heterogeneous samples (remitted vs. current, or remitted vs. non-remitting), which left them underpowered. This is important because not all individuals with LLD have MCI or develop dementia. In the few studies that included individuals with MCI or LLD plus MCI, the effects of LLD plus MCI were larger than the independent effects of these disorders, suggesting that the effects of LLD were magnified by comorbid cognitive impairment, and vice versa. This pattern is in line with our hypothesis and was implicated in previous papers as supporting the association between LLD and dementia (Butters et al., 2008; Bhalla et al., 2009; Wu et al., 2011; Andreescu et al., 2013; Boccia et al., 2015; Diniz et al., 2015, 2016; Smagula et al., 2015).

Another theme that emerged from our review is that an increasing number of studies are using network and graph theory analyses especially for comparisons among more than two groups. Four out of seven studies with additional comparison groups investigated whole brain topology in LLD, aMCI, and LLD+aMCI (Bai et al., 2012; Li et al., 2015a; Chen et al., 2016; Mai et al., 2017). These studies found disrupted network topologies—including reduced network strength, global efficiency and higher modularity- in individuals with LLD, LLD+MCI, and aMCI in comparison to HC. In addition to these differences in the global network topology, hub regions with significant diagnosis effects were mainly impaired in the frontal-executive and corticolimbic circuits. Results from network and graph theory analyses suggest that in addition to the alteration of frontal-executive and corticolimbic circuits, impairment of global brain topology is present in these disorders. Therefore, it is possible that some of the neuroimaging measurement strategies were not sufficiently addressing the underlying neurocircuit disruption, e.g., through a focus only on specific white matter tracts. Network-based analyses of brain structure and function may more directly index subtle disruptions in individuals with LLD, or LLD+MCI.

Finally, posterior DMN (posterior cingulate cortex and precuneus) appeared to have the most structural and functional alterations in LLD, and to a greater extent in aMCI and LLD+aMCI. For instance, in a DWI study, individuals with MCI had lower nodal efficiency in the posterior cingulate cortex compared to individuals with LLD (Bai et al., 2012). In rs-fMRI studies, significant ReHo reduction was detected in the posterior DMN in individuals with LLD (Yuan et al., 2008; Chen et al., 2012). In a unique longitudinal study, left hippocampal functional connectivity had a greater decline in bilateral posterior DMN in individuals with LLD compared to the HCs (Wang et al., 2015). Individuals with LLD+aMCI demonstrated lower functional connectivity in the posterior DMN relative to those with LLD (Yue et al., 2013; Li et al., 2015b). Posterior cingulate cortex is a critical brain region, and a marker of very early progression of AD as seen with T1-weighted imaging, functional MRI, postmortem, and PET studies (Rami et al., 2012; Scheff et al., 2015; Mutlu et al., 2016). These specific DMN nodes may be important as potential treatment targets in delaying the progression of LLD or LLD+MCI to dementia.

### Limitations

The heterogeneity among individuals with depression as mentioned before (including early-onset depression vs. late-onset depression, and active vs. remitted, treatment-responsive vs. treatment-resistant), and medication effects including antidepressant and cognitive enhancer agents are factors that should carefully be controlled for in future studies. Hence, in the current study, we could not evaluate how different ages of onset, remission status of depression, medication types, or response to medications may influence circuit disruption. Some T1-weighted imaging studies have reported differences between early-onset depression and late-onset depression (Ballmaier et al., 2008; Sachs-Ericsson et al., 2013; Lebedeva et al., 2015), however further exploration in all imaging modalities is required. Moreover, one third of the studies have only used MMSE to measure cognition which lacks sensitivity to subtle changes in cognition and has shown to be influenced by ceiling and floor effects (Tombaugh and McIntyre, 1992). Thus, the confounding effect of cognitive impairment in individuals with LLD in these studies may contribute to the heterogeneity of results. Furthermore, it has been shown that depression increases the risk for developing other types of dementia including vascular dementia (VaD) (Diniz et al., 2013) and Dementia with Lewy bodies (DLB) (Fujishiro, 2019; Ishiguro et al., 2019). Thus, a detailed assessment of multiple types of dementia is required in future longitudinal studies.

### Other MRI Modalities

There are other MRI modalities that were not addressed in this systematic review; T1-weighted image, T2-weighted Flair, and task-based fMRI. A meta-analysis assessed gray matter atrophy in 25 studies comparing AD and HC, and 6 studies comparing LLD and HC. It found gray matter volume reduction of the bilateral hippocampus in both individuals with LLD and AD, greater atrophy in frontal cortex in LLD, and greater atrophy of bilateral posterior cingulate cortex in AD. Two other meta-analyses of 17 VBM studies comparing LLD with HC (Sexton et al., 2013; Du et al., 2014). Sexton et al. (2013) found significant but small effect size of hippocampal volume reduction driven by seven studies, in addition to volume loss in the frontal cortex, putamen, and thalamus. Du et al. (2014) included 11 VBM studies and reported gray matter volume loss in parahippocampal gyrus, amygdala, and frontal cortex.

T2-weighted FLAIR imaging makes the detection of white matter hyperintensities (WMH) easier. WMH of presumed vascular origin is a common finding in individuals with cognitive impairment, stroke, or dementia (Wardlaw et al., 2015). Although a meta-analysis of 30 studies, reported significantly higher odds of WMH in LLD compared to HC, particularly in LOD vs. HC and EOD (Herrmann et al., 2008), recent studies have found no difference in WMH between older individuals with LLD and HCs (Harada et al., 2016) or between LLDs and aMCIs (Liao et al., 2017). Two longitudinal studies found no difference in WMH between individuals with remitted LLD and an HC group (Weber et al., 2012; Taylor et al., 2014), or between individuals with relapsed or non-remitted LLD, and an HC group (Taylor et al., 2014).

Finally, task fMRI has been used to study the functional activities and cognitive behaviors. With memory tasks, individuals with LLD, compared to HCs, demonstrated less activation of temporal lobe, hippocampus, amygdala; less functional connectivity between posterior cingulate cortex and medial temporal lobe; and enhanced activation in frontal cortex (inferior frontal gyrus, frontal pole, middle frontal gyrus) (Lee T. W. et al., 2013; Wu et al., 2013; Weisenbach et al., 2014). With executive function tasks, individuals with LLD showed deactivation in the dorsolateral prefrontal cortex, and lower functional connectivity between dorsolateral prefrontal cortex and dorsal anterior cingulate cortex, but higher BOLD response in frontal cortex (superior frontal cortices and left orbitofrontal cortex) (Aizenstein et al., 2009; Bobb et al., 2012). A meta-analysis of task-fMRI studies in individuals with MCI and AD found hypoactivation of the temporal lobe and compensatory hyperactivation in cingulate gyrus in both groups compared to the HC group (Li H. J. et al., 2015).

### Conclusion and Future Directions

Taken together, findings of the 37 reviewed studies are partially supportive of structural and functional brain alterations in both frontal-executive and corticolimbic circuits, as well as whole brain topology in LLD. Future research should focus on investigating the longitudinal effect of depression and risk of multiple types of dementia including vascular (VaD), Lewy body (DLB), and Alzheimer's dementia (AD) using multi-imaging modalities and in large groups of individuals with LLD, never-depressed MCI and LLD+MCI. This information can help to elucidate the pattern of decline in individuals with a history of depression and the link with dementia and warrants further research. Thus, future studies should include milder and more severe depression cases, as well as cases in remission vs. cases who are actively ill, use multimodal imaging techniques and consider using sophisticated analysis methods in large enough sample sizes to determine which subgroups share risk in impaired brain circuit with MCI and dementia. Such a study is now in progress, funded by the NIMH. It aims to recruit 750 older individuals with treatment-resistant LLD, who are receiving standardized treatment as part of a pragmatic clinical trial. This longitudinal study will determine who among remitters vs. non-remitters (those with persistent depression), are at highest risk of neural circuit and cognitive change (including progression to dementia over a 24-month period). The specific aims are: (1) to test whether persistent (non-remitting) depression leads to greater cognitive decline focusing on executive and episodic memory cognitive domains and greater degradation of neural circuit crucial for effective for such cognitive functions; and (2) to test whether greater degradation of neural circuit is associated with greater cognitive decline. An exploratory aim is to build a data-driven [demographic, clinical, cognitive, imaging, senescence-associated secretory phenotype (SASP) index] model using multivariate learning methods to distinguish, at baseline, who is at greatest risk for cognitive decline; and, to analyze latent class trajectories of depressive symptoms to go beyond the dichotomy of remission/non-remission to identify subsets of individuals at highest risk of cognitive decline, neural circuit change, and progression to dementia.

## Data Availability Statement

The raw data supporting the conclusions of this article will be made available by the authors, without undue reservation, to any qualified researcher.

## Author Contributions

NR-R: substantial contributions to the conception or design of the work, or the acquisition, analysis or interpretation of data for the work, drafting the work or revising it critically for important intellectual content, and agree to be accountable for all aspects of the work in ensuring that questions related to the accuracy or integrity of any part of the work are appropriately investigated and resolved. DM: substantial contributions to the conception or design of the work, or the acquisition of data for the work. MB and BM: substantial contributions to the conception or design of the work, revising the work critically for important intellectual content, and provide approval for publication of the content. AV: substantial contributions to the conception or design of the work, revising the work critically for important intellectual content, provide approval for publication of the content, and agree to be accountable for all aspects of the work in ensuring that questions related to the accuracy or integrity of any part of the work are appropriately investigated and resolved.

## Conflict of Interest

The authors declare that the research was conducted in the absence of any commercial or financial relationships that could be construed as a potential conflict of interest.
