# Supplementary material for: Evidence for Structural and Functional Alterations of Frontal-Executive and Corticolimbic Circuits in Late-Life Depression and Relationship to Mild Cognitive Impairment and Dementia: A Systematic Review
Source: Front Neurosci. 2020 Apr 17;14:253. doi: 10.3389/fnins.2020.00253 (PMC7182055; doi:10.3389/fnins.2020.00253)
Supplement: Supplementary file 1 [file Table_1.doc]

| **Reference** | **study type** | **LLD (n=)** | **HC (n=)** | **Image Modality** | **NOS** |
| --- | --- | --- | --- | --- | --- |
| (Alves *et al.* 2012) | cross-sectional | 17 | 18 | DWI | 6 |
| (Bezerra *et al.* 2012) | cross-sectional | 47 | 36 | DWI | 6 |
| (Charlton *et al.* 2014) | cross-sectional | 23 | 23 | DWI | 7 |
| (Charlton *et al.* 2015) | cross-sectional | 28 | 48 | DWI | 5 |
| (Colloby *et al.* 2011) | cross-sectional | 38 | 30 | DWI | 3 |
| (Emsell *et al.* 2017) | cross-sectional | 55 | 52 | DWI | 5 |
| (Guo *et al.* 2014) | cross-sectional | 15 | 15 | DWI | 5 |
| (Harada *et al.* 2018) | cross-sectional | 45 | 61 | DWI | 5 |
| (Li X. *et al.* 2017) | 1-year cohort | 24 | 24 | DWI | 5 |
| (Mettenburg *et al.* 2012) | cross-sectional | 51 | 16 | DWI | 5 |
| (Shimony *et al.* 2010) | cross-sectional | 73 | 23 | DWI | 4 |
| (Yuan *et al.* 2010) | cross-sectional | 37 | 33 | DWI | 7 |
| (Harada *et al.* 2016) | cross-sectional | 16 | 30 | DWI + rs-fMRI | 6 |
| (Sexton *et al.* 2012) | cross-sectional | 36 | 25 | DWI + rs-fMRI | 5 |
| (Tadayonnejad *et al.* 2014) | cross-sectional | 10 | 15 | DWI + rs-fMRI | 4 |
| (Yin *et al.* 2016a) | cross-sectional | 32 | 39 | DWI + rs-fMRI | 8 |
| (Alalade *et al.* 2011) | cross-sectional | 11 | 18 | rs-fMRI | 4 |
| (Alexopoulos *et al.* 2012) | 12-week clinical trial | 16 | 10 | rs-fMRI | 6 |
| (Chen *et al.* 2012) | cross-sectional | 17 | 16 | rs-fMRI | 5 |
| (Hou et al. 2016) | cross-sectional | 31 | 37 | rs-fMRI | 7 |
| (Ikuta *et al.* 2017) | cross-sectional | 33 | 62 | rs-fMRI | 6 |
| (Li W. et al. 2017) | cross-sectional | 39 | 29 | rs-fMRI | 6 |
| (Shu *et al.* 2014) | cross-sectional | 31 | 29 | rs-fMRI | 5 |
| (Wang *et al.* 2015) | 21-month cohort | 14 | 16 | rs-fMRI | 4 |
| (Wu, *et al.* 2011) | 12-week clinical trial | 12 | 12 | rs-fMRI | 2 |
| (Yuan *et al.* 2008) | cross-sectional | 18 | 14 | rs-fMRI | 7 |
| (Yue *et al.* 2013) | cross-sectional | 22 | 22 | rs-fMRI | 6 |
| (Yue *et al.* 2015) | cross-sectional | 16 | 16 | rs-fMRI | 4 |
| (Yin *et al.* 2015) | cross-sectional | 32 | 39 | rs-fMRI  (cerebellum seed) | 8 |
| (Yin *et al.* 2016b) | cross-sectional | 33 | 31 | rs-fMRI  (graph theory DMN) | 5 |

Supplementary Table 1. DWI and rs-fMRI studies with LLD and HC only
