# Supplementary material for: Evidence for Structural and Functional Alterations of Frontal-Executive and Corticolimbic Circuits in Late-Life Depression and Relationship to Mild Cognitive Impairment and Dementia: A Systematic Review
Source: Front Neurosci. 2020 Apr 17;14:253. doi: 10.3389/fnins.2020.00253 (PMC7182055; doi:10.3389/fnins.2020.00253)
Supplement: Supplementary file 2 [file Table_2.doc]

Supplementary Table 2. DWI and rs-fMRI studies with LLD, HC, and additional comparison groups

| **Reference** | **study type** | **LLD (n=)** | **HC (n=)** | **aMCI (n=)** | **LLD+aMCI (n=)** | **Total N** | **Image Modality** | **NOS** |
| --- | --- | --- | --- | --- | --- | --- | --- | --- |
| (Bai *et al.* 2012) | cross-sectional | 35 | 30 | 38 | - | 103 | DWI | 6 |
| (Li *et al.* 2014) | cross-sectional | 20 | 33 | 18 | 13 | 84 | DWI | 7 |
| (Mai *et al.* 2017) | cross-sectional | 24 | 30 | - | 15  (memory deficit) | 69 | DWI | 6 |
| (Li *et al.* 2015a) | cross-sectional | 23 | 25 | 18 | 13 | 79 | rs-fMRI | 7 |
| (Li *et al*. 2015b) | cross-sectional | 25 | 26 | - | 15 | 63 | rs-fMRI | 7 |
| (Xie *et al.* 2013) | cross-sectional | 18 | 25 | 12 | 12 | 72 | rs-fMRI | 7 |
| (Chen *et al.* 2016) | cross-sectional | 55 | 114 | 87 | - | 256 | rs-fMRI | 7 |
